# Adipose tissue-derived cytokines and their correlations with clinical characteristics in Vietnamese patients with type 2 diabetes mellitus

**DOI:** 10.1186/s13098-018-0343-4

**Published:** 2018-05-15

**Authors:** Nguyen Linh Toan, Nguyen Van Hoan, Doan Viet Cuong, Nguyen Viet Dung, Phan The Dung, Ngo Thu Hang, Do Thi Huyen Dieu, Dang Thanh Chung, Ho Anh Son, Pham Xuan Phong, George Binh Lenon, Doan Van De, Hoang Van Tong

**Affiliations:** 1 Department of Pathophysiology, Vietnam Military Medical University, 160 Phung Hung, Ha Dong, Hanoi, Vietnam; 2 Department of Endocrine, Vietnam Military Medical University, 103 Military Hospital, Hanoi, Vietnam; 3 Nghe An Endocrine Hospital, Nghe An, Vietnam; 4 Binh Dinh Medical School, Qui Nhon, Binh Dinh Vietnam; 5 Military Institute of Traditional Medicine, Hanoi, Vietnam; 6 Discipline of Chinese Medicine, School of Health & Biomedical Sciences, RMIT University, Melbourne, Australia; 7 Institute of Biomedicine and Pharmacy, Vietnam Military Medical University, Hanoi, Vietnam

**Keywords:** Type 2 diabetes mellitus, Adipokine, Overweight, Vietnam

## Abstract

**Background:**

Adipokines are involved in the pathogenesis of metabolic disorders including obesity and type 2 diabetes mellitus (T2DM). This study investigates the levels of leptin, resistin, visfatin, secreted frizzled-related protein 5 (SFRP5), monocyte chemoattractant protein-1 (MCP-1) and retinol-binding protein 4 (RBP4) and their correlations with clinical parameters of overweight and T2DM.

**Methods:**

We recruited overweight 50 patients with T2DM, 88 non-overweight patients with T2DM, 29 overweight and 100 non-overweight individuals devoid of T2DM for this study. The levels of studied adipokines were measured by enzyme-linked immunosorbent assay and correlated with clinical parameters.

**Results:**

The levels of MCP-1 and SFRP5 were decreased while visfatin and RBP4 levels were increased in patients with T2DM compared to those in the control individuals (*P *< 0.01). Among patients with T2DM, leptin and resistin levels were higher while RBP4 levels were lower in patients with overweight T2DM compared to those in patients with non-overweight T2DM (*P *< 0.0001, 0.019 and 0.05, respectively). Leptin and MCP-1 levels were correlated with HOMA-IR, QUICKI and HOMA-β. Leptin/MCP-1 ratio was correlated with insulin levels, HOMA-IR and HOMA-β indexes. Resistin/RBP4, visfatin/MCP-1 and MCP-1/RBP4 ratios were strongly correlated with the levels of fasting glucose, HbA1c and HOMA-β. In addition, ROC curve analyses indicated a diagnostic potential of resistin/RBP4 and MCP-1/RBP4 indexes for T2DM (AUC = 0.81 and 0.83, respectively) and β-cell function (AUC = 0.76 and 0.74, respectively).

**Conclusions:**

Adipokines (leptin, resistin, visfatin, SFRP5, MCP-1, and RBP4) are associated with overweight and T2DM and may serve as a potential prognostic marker and therapeutic intervention for overweight-related T2DM.

## Background

Type 2 diabetes mellitus (T2DM) is a chronic metabolic disorder and is exponentially increasing in developing countries including Vietnam due to an increased consumption of energy-rich food, sedentary lifestyle, and urbanization [1]. According to the International Diabetes Federation (IDF), there were 382 million diabetic cases in 2013 and the number of diabetic cases is predicted to be 592 million by the year 2035 [2, 3]. Approximately 80% of diabetic cases are in developing countries and there are 5.1 million deaths worldwide annually due to this disease [2, 3]. The prevalence of T2DM is 1.2% in developed countries while there is four times higher in developing countries [3]. In Vietnam, the prevalence of T2DM is increasing rapidly and approximately 3.3 million diabetic cases were reported in 2014. The prevalence of diabetes in the age group of 30–69 years is estimated to be 5.7% across the country and 7% in the urban areas [4, 5]. However, a recent study conducted with a large sample size in the Red River Delta of Vietnam showed that the general population aged 40–64 years had a low level of diabetes knowledge, especially in the rural areas [6]. This might be one of the challenges for public health to control the disease in Vietnam.

T2DM constitutes up to 90% of all diabetes and it is characterized by chronic hyperglycaemia resulting from defects in insulin secretion and/or insulin action and metabolic disorders of protein and lipids [7, 8]. The main pathogenesis of T2DM includes insulin resistance and insufficient insulin production of pancreatic β cells, which lead to the disability to control glucose level in the circulation [9]. Additionally, the increased production of pro-inflammatory cytokines due to obesity contributes to the increased risk of T2DM development [7, 10]. Insulin resistance is significantly related to obesity and develops years before the clinical manifestations of T2DM [11]. Adipose tissue is considered as an endocrine organ that produces and metabolites numerous proteins, hormones, cytokines, and sex steroids, namely adipocytokine (or adipokines) [12, 13]. Adipocytes are also involved in various biological processes such as energy metabolism, neuroendocrine function and immune response through the activities of their receptors for hormones of the endocrine and central nervous system [13]. The increased adipose tissue is associated with insulin resistance, elevated blood glucose levels, lipid metabolic disorders, hypertension and inflammation [14].

Adipose tissue-derived proteins with hormone activities regulate metabolic functionalities and inflammation [14]. The adipose tissue-derived adipokines such as adiponectin, leptin, visfatin, resistin and adipsin are associated with obesity and obesity-related metabolic disorders including T2DM [15]. Other adipose tissue-derived proteins also play important roles in metabolism, immune response, and metabolic disorders. Particularly, secreted frizzled-related protein 5 (SFRP5) is a member of the Sfrp family that is involved in the modulation of the Wnt signaling [16]. SFRP5 is highly expressed in adipose tissue and has been associated with β-cell function, glucose metabolism, obesity and T2DM [17–20]. Monocyte chemoattractant protein-1 (MCP-1), known as chemokine (C–C motif) ligand 2 (CCL2), is a chemoattractant protein for several immune cells including monocytes/macrophages, T cells and natural killer (NK) cells, and this protein has been implicated in adipose tissue inflammation [21]. In addition, retinol-binding protein 4 (RBP4) is an adipose tissue-derived cytokine that transports retinol from the liver to various tissues and it is related to insulin resistance, visceral fat distribution, and dyslipidemia [22, 23].

Our previous study showed that the levels of adiponectin and pro-inflammatory cytokines including tumor necrosis factor (TNF)-α, IL-1β and IL-10 are modulated and correlated with clinical parameters of overweight and T2DM in Vietnamese patients with T2DM and in overweight individuals [24]. The present study aims to investigate the levels of leptin, resistin, visfatin, SFRP5, MCP-1/CCL2, and RBP4 as well as their correlations with insulin resistance and clinical parameters of overweight and T2DM in a Vietnamese study group.

## Methods

### Study population

The study was designed as a case-controlled study combined with clinical observations and experimental analyses. One hundred and thirty-eight (n = 138) Vietnamese patients with T2DM and one hundred and twenty-nine (n = 129) control individuals were recruited during 2013 and 2014 for this study (Table 1).

**Table 1:** Characteristics of patients with type 2 diabetes mellitus and controls

Characteristics	Type 2 diabetes mellitus			Without type 2 diabetes mellitus			*P* value (*)
	Overweight T2DM (n = 50)	Non-overweight T2DM (n = 88)	*P* value	Overweight individuals (n = 29)	Non-overweight individuals (n = 100)	*P* value	
Demographics							
Age (years)	58 [40–84]	59 [40–69]	NS	59 [35–80]	57 [37–70]	NS	NS
Gender (M/F)	29/21	50/38	NS	13/16	30/70	NS	0.0001
Smoking (yes/no)	5/45	14/74	NS	2/27	4/96	NS	0.011
Alcohol usage (yes/no)	15/35	26/62	NS	6/23	6/94	0.027	< 0.0001
Physical exercise (yes/no)	35/15	73/15	NS	16/13	82/18	0.006	NS
Family history of T2DM (yes/no)	3/47	11/77	NS	2/27	1/99	NS	0.009
Anthropometrics							
WHR	0.94 [0.86–1.07]	0.92 [0.83–1.18]	< 0.0001	0.93 [0.82–1.1]	0.88 [0.72–1.3]	0.001	< 0.0001
BMI	26.7 [25–36.3]	22.4 [17.7–24.9]	< 0.0001	26.2 [25.1–29.6]	22.3 [16–24.9]	< 0.0001	0.008
Biochemical and clinical characteristics							
GOT (AST) (U/l)	22 [10–60]	19 [10–65]	0.025	27 [19–46]	24 [4–105]	0.043	0.006
GPT (ALT) (U/l)	28 [11–109]	19.5 [8–86]	0.001	29 [19–68]	21 [7–177]	0.001	NS
Total bilirubin (µmol/l)	9.4 [4.3–21.2]	7.5 [3.4–21.6]	0.047	9.4 [4.4–18.7]	9.8 [2.7–30]	NS	0.001
Blood urea (mmol/l)	5.2 [3.2–9]	5.4 [2.1–11.8]	NS	5.5 [3.6–8.7]	5.65 [2.7–9.2]	NS	NS
Blood creatinine (µmol/l)	69.5 [38–123]	68 [38–121]	NS	67 [48–128]	64 [45–96]	NS	0.015
Fasting glucose (mmol/l)	8.6 [7–28.9]	7.65 [7–21.6]	0.002	5.3 [4.3–5.6]	5.3 [4–5.6]	NA	< 0.0001
Urine glucose (pos/neg)	10/40	12/76	NS	0/29	6/94	NA	0.0026
Triglycerides (mmol/l)	2.5 [0.4–9.1]	1.8 [0.34–19.6]	0.014	1.97 [0.5–6.0]	1.43 [0.3–16.8]	NS	0.023
Total cholesterol (mmol/l)	5.0 [2.9–9.2]	5.0 [3.8–8.5]	NS	4.6 [3.3–7.3]	5.2 [3.0–7.8]	0.012	NS
HDL-C (mmol/l)	1.1 [0.6–1.85]	1.2 [0.7–4.6]	0.042	1.26 [0.9–2.1]	1.35 [0.7–2.7]	0.019	0.002
LDL-C (mmol/l)	2.7 [0.8–4.1]	3.0 [1.0–4.5]	NS	2.7 [0.8–4.8]	3.0 [1.1–5.9]	0.037	NS
Glycosylated hemoglobin (HbA1c) (%)	7 [5.4–14.4]	6.8 [5.2–10.9]	NS	5.9 [4–6.4]	5.6 [4.2–8.5]	0.004	< 0.0001
Insulin (mIU/l)	8.96 [2.7–54.5]	6.5 [1.3–23.2]	0.002	7.8 [3.5–10.2]	6.4 [0.08–17.5]	NS	0.004
HOMA-IR	3.6 [1.3–48.9]	2.3 [0.4–9.7]	< 0.0001	1.8 [0.8–2.5]	1.5 [0.01–4.2]	NS	< 0.0001
QUICKI	0.8 [0.5–0.9]	0.86 [0.7–1.07]	< 0.0001	0.9 [0.8–1.4]	0.9 [0.7–1.6]	NS	< 0.0001
HOMA-β	33.6 [3.5–279.2]	27.4 [4.6–111.5]	NS	89.9 [38.5–222.1]	74.1 [3.0–370.8]	NS	< 0.0001

*T2DM* type 2 diabetes mellitus, *BMI* body mass index, *WHR* waist-to-hip ratio, *GOT* Glutamic-Oxaloacetic Transaminase (AST), *GPT* Glutamic-Pyruvic Transaminase (ALT), *HDL-C* high-density lipoprotein-cholesterol, *LDL-C* low-density lipoprotein-cholesterol, *HOMA-IR* Homeostasis Model Assessment-Insulin Resistance, *QUICKI* Quantitative Insulin Sensitivity Check Index, *HOMA-β* homeostatic model assessment-β-cell function, *NA* not applicable

Values given are medians and range

(*) Comparison between patients with type 2 diabetes mellitus and control individuals. *P* values were calculated by Chi square, Fisher’s exact tests or multi-factor ANOVA, where appropriate

Patients with T2DM were diagnosed based on the standard criteria reported by the World Health Organization (WHO) and by the International Diabetes Federation (IDF) [25, 26]. The inclusion and exclusion criteria for the patient group as well as the selection criteria for the control groups were described in our previous study [24]. At the time of sampling, most of the recruited patients with T2DM were newly diagnosed and had not been treated with any anti-diabetic drug. A small number of patients were immediately treated with the diabetes drug Diamicron MR and/or a low dose of insulin injection. Clinical examinations and biochemical tests for the function of the heart, lung, liver and kidney were performed as the routine. History of diabetes-associated symptoms, personal and family history of diabetes, family history of premature cardiovascular diseases, smoking, alcohol consumption, and lifestyle were obtained from all participants using the standard study questionnaires (Table 1). The anthropometric data such as height, weight, waist and hip circumferences were measured using standard procedures for all study participants. Body mass index (BMI) and waist-hip ratio (WHR) were calculated based on their anthropometric measurements. Based on their BMI and T2DM status, the patient group was divided into two subgroups: overweight (BMI ≥ 25) with T2DM (n = 50) and non-overweight (BMI < 25) with T2DM (n = 88). Similarly, the control group was also divided into two subgroups including overweight control individuals (BMI ≥ 25; n = 29) and non-overweight control individuals (BMI ranging from 16 to 25) without T2DM (n = 100) (Table 1).

### Measurement of biochemical parameters

The levels of lipid components including cholesterol (CT), triglycerides (TG), high-density lipoprotein-cholesterol (HDL-C), low-density lipoprotein-cholesterol (LDL-C), fasting glucose, insulin and blood fasting glycosylated hemoglobin (HbA1c) were routinely measured as described previously [24]. Based on the levels of insulin and glucose, the homeostasis model assessment insulin resistance (HOMA-IR) index were calculated using formulas HOMA-IR = glucose × insulin/22.5; the quantitative insulin sensitivity check index (QUICKI) [QUICKI = 1/(log(fasting insulin) + log(fasting glucose))] and the homeostatic model assessment-beta (HOMA-β) [HOMA-IR = (20 × insulin)/(glucose-3.5)]. These indexes were calculated to evaluate the levels of insulin resistance/sensitivity and β-cell function [27, 28].

### Measurement of leptin, resistin, visfatin, SFRP5, MCP-1, and RBP4 levels

The levels of leptin, resistin, visfatin, SFRP5, MCP-1 and RBP4 were measured in the respective serum samples of the study participants by using commercially available ELISA kits following the manufacturer’s instruction. The ELISA kits for leptin (Cat. No.: DEE007), resistin (Cat. No.: DEE050), and for visfatin (Cat. No.: DERAG004R) were purchased from Demeditec Diagnostics GmbH, Kiel, Germany. The ELISA kits for MCP-1 (Cat. No.: EH2MCP1) was purchased from Thermo Scientific, PO Box 117 Rockford, IL61105, United States. The ELISA kits for SFRP5 (Cat. No.: DY6266-05) was purchased from R&D System, 614 McKinley Place NE, Minneapolis, MN 55413, United States. The EIA kit for RBP4 (Cat. No.: RAB0414-1KT) was purchased from Sigma-Aldrich, Missouri, the United States. The detection limit of the ELISA kits is 0.2 ng/ml for leptin, 0.012 ng/ml for resistin, 0.125 ng/ml for visfatin, 15.6 pg/ml for SFRP5, 0 pg/ml for MCP-1, and 0.1 pg/ml for RBP4.

### Statistical analysis

Clinical and demographic data were presented in median values with range for continuous variables. Chi square or Fisher’s exact tests were used to compare categorical variables. Multi-factor ANOVA was performed for comparing means between groups and to adjust for the confounding effect of age and sex on the clinical parameters and investigated adipokines. Kruskal–Wallis, Mann–Whitney U test was used to analyze the serum levels of leptin, resistin, visfatin, SFRP5, MCP-1, and RBP4 in patients with T2DM and in controls wherever appropriate. Spearman’s rank correlation coefficient was used to analyze the correlation between two studied variables. The diagnostic potential of investigated adipose tissue-derived cytokines and their relative ratios in T2DM, insulin resistance/sensitivity and β-cell function was evaluated by receiver operating characteristic (ROC) analysis and the area under the curve (AUC) was also calculated. All statistical analyses were performed using IBM Statistics SPSS v.19 (IBM Corp, Armonk, NY. the USA). *P* values of less than 0.05 were considered significant.

## Results

### Demographic, anthropometric, biochemical and clinical characteristics of the study subjects

The demographic, anthropometric, biochemical and clinical characteristics of the patients with T2DM and control individuals are summarized in Table 1. No statistical differences between the mean age of study group (with T2DM, 58.6 ± 7.2 years) and control group (without T2DM; 56.8 ± 8.2 years), between patients with overweight T2DM and those with non-overweight T2DM, as well as between control individuals with and without overweight (*P *> 0.05) were observed. Regarding gender, there was a higher number of male participants in the group with T2DM compared to that in control group (*P *= 0.0001). The proportions of smokers and alcohol users in the patient group were also higher compared to that in control group (*P *= 0.001 and < 0.0001, respectively). In addition, the proportion of patients with T2DM whose at least one of their direct relatives (parents, siblings, children) had T2DM was higher compared to that of the control group (*P *= 0.009). These results demonstrate that the smoking, alcohol usage and family history of T2DM are associated with the prevalence of T2DM (Table 1).

The levels of fasting glucose, triglycerides and glycosylated hemoglobin (HbA1c) were higher in patients with T2DM compared to those in control individuals (*P *< 0.0001, 0.023, and < 0.0001, respectively). Glucose was detected more frequently in urine samples of patients with T2DM compared to that in the control group (*P *= 0.0026). The levels of high-density lipoprotein-cholesterol (HDL-C) were lower in patients with T2DM compared to those in control individuals (*P *= 0.002). However, no significant difference in the levels of low-density lipoprotein-cholesterol (LDL-C), insulin and total cholesterol between the two groups was observed (*P *> 0.05). HOMA-IR and HOMA-β indexes were higher while QUICKI index was lower in patients with T2DM compared to those in the control group (*P *< 0.0001) (Table 1).

Within the group of patients with T2DM, the levels of fasting glucose and triglycerides were higher while HDL-C levels were decreased in the patients with overweight T2DM compared to those with non-overweight T2DM (*P *= 0.002, 0.014, 0.042, respectively). Insulin levels and HOMA-IR index were higher while QUICKI index was lower in patients with overweight T2DM compared to those with non-overweight T2DM (*P *= 0.04, 0.028 and 0.016, respectively). However, within the control individuals, insulin levels, HOMA-IR, HOMA-β and QUICKI indexes were not significantly different between control individuals with and without overweight (*P *> 0.05). In addition, no difference in the levels of fasting glucose and triglycerides was observed between the two control subgroups (*P *> 0.05) (Table 1).

### Levels of leptin, resistin, visfatin, SFRP5, MCP-1, and RBP4

**Fig. 1:**
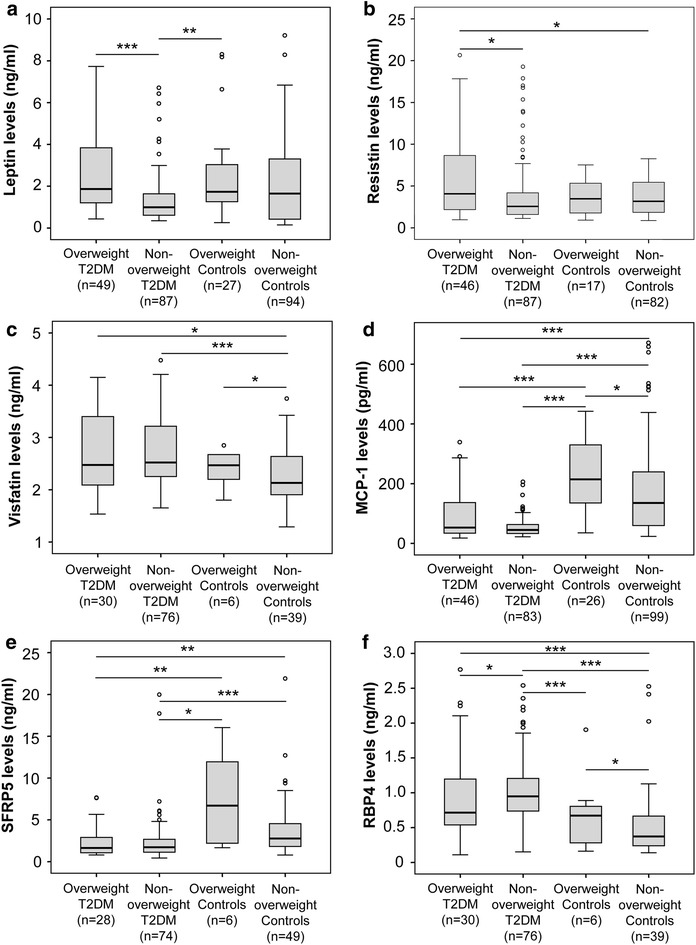
The levels of studied adipokines in patients with type 2 diabetes mellitus and in control individuals. The levels of leptin (**a**), resistin (**b**), visfatin (**c**), monocyte chemoattractant protein-1 (MCP-1) (**d**), secreted frizzled-related protein 5 (SFRP5) (**e**), and retinol binding protein 4 (RBP4) (**f**) were measured in the serum samples from patients with overweight and non-overweight T2DM, as well as in overweight and non-overweight control individuals. *P* values were calculated by using Mann–Whitney U test; *P < 0.05; **P < 0.005; ***P < 0.0005

The levels of MCP-1 and SFRP5 were significantly decreased (*P *< 0.0001 and *P *= 0.003, respectively), while the levels of visfatin and RBP4 were significantly increased in patients with T2DM compared to those in control individuals (*P *= 0.005 and *P *= 0.021, respectively). However, no difference in the levels of leptin and resistin between the two groups was observed (*P *> 0.05) (Fig. 1). Among the patients with T2DM, the levels of leptin and resistin were higher while the RBP4 levels were lower in patients with overweight T2DM compared to those in patients with non-overweight T2DM (*P *= 0.0001, *P *= 0.019, and *P *= 0.05, respectively). Among the control group, the levels of visfatin, MCP-1 and RBP4 were higher in overweight individuals compared to those in non-overweight controls (*P *= 0.022, 0.048 and 0.05, respectively). We also observed a similar trend for leptin, resistin, and SFRP5 between overweight and non-overweight control subgroups, but the difference did not reach the statistical significance. In addition, we observed a difference of MCP-1, SFRP5 and RBP4 levels between overweight with T2DM and overweight control groups as well as between non-overweight with T2DM and non-overweight control groups (*P *< 0.05) (Fig. 1).

### Correlation between studied adipokines

We analyzed the correlations between pairs of the studied adipokine levels. We observed that MCP-1 levels were positively correlated with the levels of resistin and visfatin (Spearman’s rho = 0.22, *P *= 0.001 and rho = 0.29, *P *< 0.0001, respectively). Visfatin levels were correlated with SFRP5 levels (Spearman’s rho = 0.24, *P *= 0.015). RBP4 levels were negatively correlated with resistin and MCP-1 levels (Spearman’s rho = − .22 *P *= 0.001 for both resistin and MCP-1). In addition, we observed weak correlations between leptin and resistin (Spearman’s rho = 0.18, *P *= 0.009), leptin and SFRP5 (Spearman’s rho = 0.17, *P *= 0.037), resistin and SFRP5 (Spearman’s rho = 0.17, *P *= 0.038), as well as between MCP-1 and SFRP5 (Spearman’s rho = 0.18, *P *= 0.025) (Table 2).

**Table 2:** Correlation between pair of studied adipokines

Adipokines	Leptin (ng/ml)		Resistin (ng/ml)		Visfatin (ng/ml)		MCP-1 (pg/ml)		SFRP5 (ng/ml)		RBP4 (ng/ml)	
	ρ (rho)	*P* value	ρ (rho)	*P* value	ρ (rho)	*P* value	ρ (rho)	*P* value	ρ (rho)	*P* value	ρ (rho)	*P* value
Leptin (ng/ml)			0.18	0.009	− 0.004	0.96	0.09	0.15	0.17	0.037	− 0.03	0.63
Resistin (ng/ml)	0.18	0.009			− 0.02	0.77	*0.22*	*0.001*	0.17	0.038	− *0.22*	*0.001*
Visfatin (ng/ml)	− 0.004	0.96	− 0.02	0.77			*0.29*	*<* *0.0001*	*0.24*	*0.015*	− 0.12	0.18
MCP-1 (pg/ml)	0.09	0.15	*0.22*	*0.001*	*0.29*	*<* *0.0001*			0.18	0.025	− *0.22*	*0.001*
SFRP5 (ng/ml)	0.17	0.037	0.17	0.038	*0.24*	*0.015*	0.18	0.025			0.05	0.56
RBP4 (ng/ml)	− 0.03	0.63	− *0.22*	*0.001*	− 0.12	0.18	− *0.22*	*0.001*	0.05	0.56		

The correlations between pair of studied adipokines were calculated by using the Spearman’s rank correlation coefficient. Spearman’s rho (ρ) and *P* values are presented

### Correlation of leptin, resistin, visfatin, SFRP5, MCP-1, and RBP4 levels with metabolic parameters

We analyzed a correlation of the levels of the studied adipokines with the metabolic parameters of overweight and T2DM. We observed that the leptin levels were positively correlated with insulin levels (Spearman’s rho = 0.43, *P *< 0.0001), HOMA-IR (Spearman’s rho = 0.31, *P *< 0.0001), HOMA-β (Spearman’s rho = 0.3, *P *< 0.0001) and negatively correlated with QUICKI indexes (Spearman’s rho = − 0.29, *P *< 0.0001). Resistin levels were positively correlated with HOMA-β (Spearman’s rho = 0.33, *P *< 0.0001) and negatively correlated with the levels of fasting glucose and HbA1c (Spearman’s rho = − 0.32, *P *< 0.0001, and rho = − 0.29, *P *< 0.0001, respectively). Visfatin levels were weakly and negatively correlated with fasting glucose levels (Spearman’s rho = − 0.22, *P *= 0.006). MCP-1 levels were positively correlated with the QUICKI (Spearman’s rho = 0.26, *P *< 0.0001) and HOMA-β (Spearman’s rho = 0.33, *P *< 0.0001) indexes, and negatively correlated with the fasting glucose levels (Spearman’s rho = − 0.42, *P *< 0.0001), HbA1c (Spearman’s rho = − 0.35, *P *< 0.0001) and HOMA-IR (Spearman’s rho = − 0.25, *P *< 0.0001). SFRP5 levels were positively correlated with the levels of LDL-C levels (Spearman’s rho = 0.28, *P *< 0.0001) and HOMA-β index (Spearman’s rho = 0.21, *P *= 0.007), and negatively correlated with fasting glucose levels (Spearman’s rho = − 0.25, *P *= 0.001). In addition, RBP4 levels were positively correlated with the levels of fasting glucose (Spearman’s rho = 0.4, *P *< 0.0001) and HbA1c (Spearman’s rho = 0.41, *P *< 0.0001), and negatively correlated with HOMA-β index (Spearman’s rho = − 0.37, *P *< 0.0001) (Table 3).

**Table 3:** Correlation between studied adipokines and clinical parameters of obesity and type 2 diabetes mellitus

Clinical characteristics	Leptin (ng/ml)		Resistin (ng/ml)		Visfatin (ng/ml)		MCP-1 (pg/ml)		SFRP5 (ng/ml)		RBP4 (ng/ml)	
	ρ (rho)	*P* value	ρ (rho)	*P* value	ρ (rho)	*P* value	ρ (rho)	*P* value	ρ (rho)	*P* value	ρ (rho)	*P* value
Fasting glucose (mmol/l)	− 0.035	0.57	− *0.32*	*<* *0.0001*	− *0.22*	0.006	− *0.42*	*<* *0.0001*	− *0.25*	*0.001*	*0.4*	*<* *0.0001*
Triglycerides (mmol/l)	0.13	0.043	− 0.13	0.048	0.12	0.15	− 0.02	0.75	− 0.05	0.54	0.16	0.038
Total cholesterol (mmol/l)	0.17	0.006	0.014	0.84	0.1	0.2	0.005	0.94	0.14	0.092	− 0.02	0.76
HDL-C (mmol/l)	− 0.007	0.91	0.082	0.21	− 0.03	0.71	0.065	0.3	0.051	0.53	− 0.16	0.015
LDL-C (mmol/l)	0.15	0.02	0.095	0.15	− 0.002	0.98	− 0.02	0.76	*0.28*	*<* *0.0001*	− 0.04	0.57
Glycosylated hemoglobin (HbA1c) (%)	0.03	0.64	− *0.29*	*<* *0.0001*	− 0.17	0.34	− *0.35*	*<* *0.0001*	− 0.18	0.023	*0.41*	*<* *0.0001*
Insulin (mIU/l)	*0.43*	*<* *0.0001*	0.05	0.47	− 0.03	0.68	− 0.02	0.71	− 0.03	0.73	− 0.07	0.29
HOMA-IR	*0.31*	*<* *0.0001*	− 0.14	0.34	− 0.15	0.064	− *0.25*	*<* *0.0001*	− 0.17	0.031	0.14	0.029
QUICKI	− *0.29*	*<* *0.0001*	0.13	0.057	0.17	0.038	*0.26*	*<* *0.0001*	0.19	0.019	− 0.14	0.029
HOMA-β	*0.3*	*<* *0.0001*	*0.33*	*<* *0.0001*	0.14	0.084	*0.33*	*<* *0.0001*	*0.21*	*0.007*	*−* *0.37*	*<* *0.0001*

The correlations between studied adipokines and clinical parameters of overweight and T2DM were calculated by using Spearman’s rank correlation coefficient. Spearman’s rho (ρ) and *P* values are presented

*HDL-C* high-density lipoprotein-cholesterol, *LDL-C* low-density lipoprotein-cholesterol, *HOMA-IR* Homeostasis Model Assessment-Insulin Resistance, *QUICKI* Quantitative Insulin Sensitivity Check Index, *HOMA-β* homeostatic model assessment-β-cell function

### Correlation of studied adipokines ratios with metabolic parameters

An index based on the relative proportion of adiponectin-to-resistin has been proposed to have diagnostic potential for insulin resistance [29]. Due to the correlation between the levels of resistin and MCP-1, resistin and RBP4, visfatin and MCP-1, visfatin and SFRP5, as well as between the levels of MCP-1 and RBP4, the relative proportion of these studied adipokines may have a potential for the diagnosis of T2DM. We calculated the relative ratios of resistin/MCP-1, resistin/RBP4, visfatin/MCP-1, visfatin/SFRP5 and MCP-1/RBP4 and analyzed their correlations with metabolic parameters (Table 4). The results showed that resistin/RBP4 relative ratio were negatively correlated with fasting glucose levels (Spearman’s rho = − 0.47, *P *< 0.0001) and positively correlated with HbA1c (Spearman’s rho = 0.43, *P *< 0.0001) and HOMA-β index (Spearman’s rho = 0.45, *P *< 0.0001). Visfatin/MCP-1 relative ratio were positively correlated with fasting glucose levels (Spearman’s rho = 0.35, *P *< 0.0001) and HbA1c (Spearman’s rho = 0.23, *P *= 0.004) and negatively correlated with HOMA-β index (Spearman’s rho = − 0.25, *P *= 0.002). MCP-1/RBP4 relative ratio were positively correlated with fasting glucose levels (Spearman’s rho = 0.51, *P *< 0.0001) and HbA1c (Spearman’s rho = 0.44, *P *< 0.0001) and negatively correlated with QUICKI index (Spearman’s rho = − 0.21, *P *= 0.002) and HOMA-β index (Spearman’s rho = − 0.46, *P *< 0.0001) (Table 4). Although leptin levels were not correlated with the levels of other adipokines, leptin and MCP-1 levels were strongly correlated with the insulin resistance/sensitivity indexes. Therefore, we calculated the relative ratio of leptin/MCP-1 and correlated with insulin resistance/sensitivity indexes as well as with other metabolic parameters. We observed that leptin/MCP-1 relative ratios were positively correlated with the levels of fasting glucose, insulin and HOMA-IR index (Spearman’s rho = 0.2, 0.31 and 0.35; *P *= 0.001, < 0.0001 and < 0.0001, respectively) and negatively correlated with QUICKI index (Spearman’s rho = − 0.35, *P *< 0.0001) (Table 4).

**Table 4:** Correlation between the relative ratios of studied cytokines and clinical parameters of obesity and type 2 diabetes mellitus

Clinical characteristics	Resistin/MCP-1		Resistin/RBP4		Visfatin/MCP-1		Visfatin/SFRP5		MCP-1/RBP4		Leptin/MCP-1	
	ρ (rho)	*P* value	ρ (rho)	*P* value	ρ (rho)	*P* value	ρ (rho)	*P* value	ρ (rho)	*P* value	ρ (rho)	*P* value
Fasting glucose (mmol/l)	0.01	0.88	− *0.47*	*< * *0.0001*	*0.35*	*<* * 0.0001*	0.1	0.29	*0.51*	*<* * 0.0001*	*0.2*	*0.001*
Triglycerides (mmol/l)	− 0.08	0.23	− 0.19	0.006	0.1	0.2	0.12	0.2	0.09	0.16	0.08	0.17
Total cholesterol (mmol/l)	− 0.07	0.3	− 0.02	0.8	− 0.03	0.7	− 0.09	0.32	0.05	0.48	0.12	0.55
HDL-C (mmol/l)	− 0.004	0.95	0.12	0.08	− 0.1	0.24	− 0.17	0.08	− 0.07	0.28	− 0.03	0.66
LDL-C (mmol/l)	0.008	0.9	0.06	0.4	− 0.02	0.78	− *0.22*	*0.019*	0.01	0.8	0.13	0.035
Glycosylated hemoglobin (HbA1c) (%)	− 0.04	0.58	0.43	< *0.0001*	*0.23*	*0.004*	0.03	0.7	*0.44*	*<* * 0.0001*	0.17	0.008
Insulin (mIU/l)	0.05	0.43	0.08	0.24	− 0.04	0.61	0.03	0.79	− 0.08	0.21	*0.31*	< *0.0001*
HOMA-IR	0.04	0.59	− 0.17	0.014	0.14	0.084	0.083	0.39	0.19	0.003	*0.35*	*<* *0.0001*
QUICKI	− 0.05	0.44	0.17	0.014	− 0.16	0.055	− 0.08	0.41	− *0.21*	*0.002*	*− 0.35*	*<* *0.0001*
HOMA-β	0.06	0.35	*0.45*	*<* *0.0001*	− *0.25*	*0.002*	− 0.1	0.31	− *0.46*	*<* * 0.0001*	0.02	0.72

Relative ratios of resistin/MCP-1, resistin/RBP4, visfatin/MCP-1, visfatin/SFRP5, MCP-1/RBP4, leptin/MCP-1 were calculated and correlated with insulin levels, and HOMA-IR, QUICKI and HOMA-β indexes. The correlations were calculated by using Spearman’s rank correlation coefficient. Spearman’s rho (ρ) and *P* values are presented

*HOMA-IR* Homeostasis Model Assessment-Insulin Resistance, *QUICKI* Quantitative Insulin Sensitivity Check Index, *HOMA-β* homeostatic model assessment-β-cell function, *HDL-C* high-density lipoprotein-cholesterol, *LDL-C* low-density lipoprotein-cholesterol

### Diagnostic potential of adipose tissue-derived cytokines and their relative ratios

**Fig. 2:**
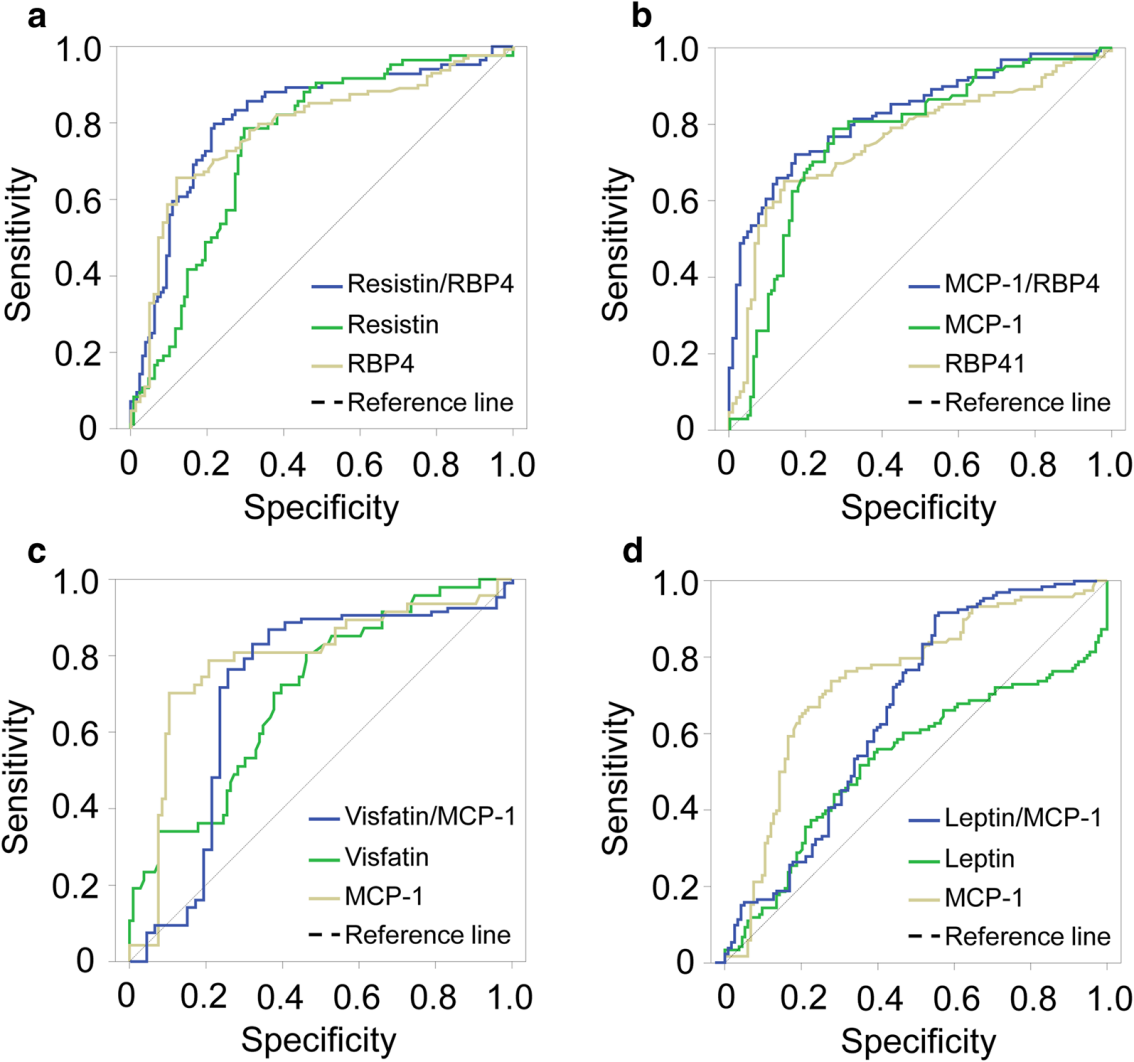
Diagnostic performance of adipose tissue-derived cytokines and their relative ratios in T2DM. ROC curves of adipose tissue-derived cytokines and their relative ratios based on the T2DM status. **a** HOMA-β versus Resistin/RBP4, Resistin and RBP4; **b** HOMA-IR versus MCP-1/RBP4, MCP-1 and RBP4; **c** HOMA-β versus Leptin/MCP-1, Leptin, and MCP-1; **d** QUICKI versus Leptin/MCP-1, Leptin, and MCP-1

**Fig. 3:**
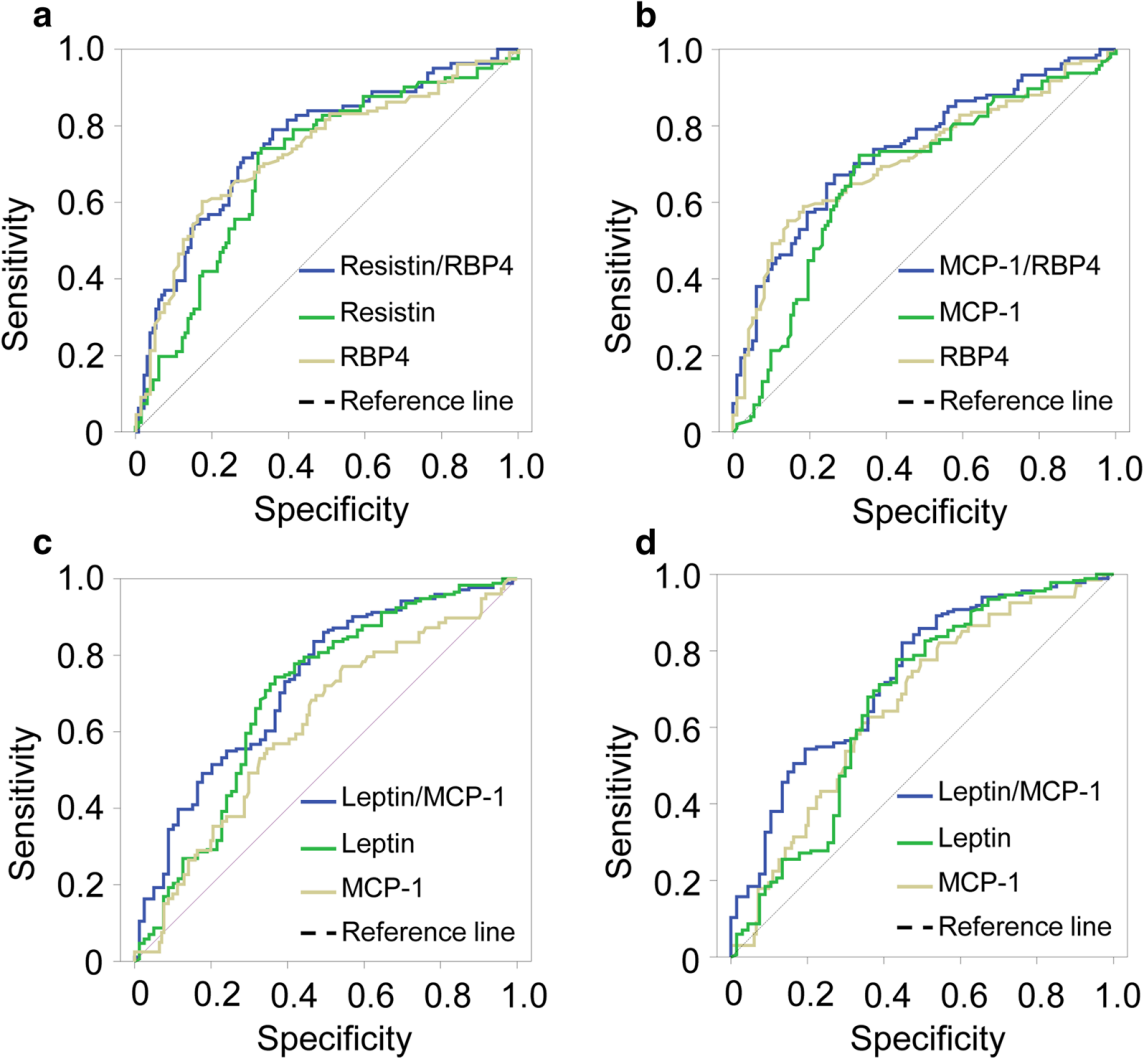
Diagnostic performance of adipose tissue-derived cytokines and their relative ratios in insulin resistance/sensitivity and β-cell function. ROC curves of adipose tissue-derived cytokines and their relative ratios based on insulin resistance/sensitivity and β-cell function. HOMA-IR, QUICKI, and HOMA-β were used as the predictor variables and the cut-off values were defined as 25% percentile, median and 75% percentile of the control group. Different cut-off values were used to analyze and the best diagnostic performance was presented. **a** diagnostic performances of resistin/RBP4 ratio, resistin and RBP4 in β-cell function with HOMA-β of 65.6 (25% percentile) as cut-off value. **b** diagnostic performances of MCP-1/RBP4 ratio, MCP-1 and RBP4 in β-cell function with HOMA-β of 65.6 (25% percentile) as cut-off value. Diagnostic performances of Leptin/MCP-1, Leptin and MCP-1 in insulin resistance/sensitivity with HOMA-IR of 1.99 (median) as cut-off value (**c**) and QUICKI of 0.93 (median) as cut-off value (**d**)

We examined the diagnostic potential of resistin/MCP-1, resistin/RBP4, visfatin/MCP-1, visfatin/SFRP5, MCP-1/RBP4 and leptin/MCP-1 indexes in T2DM by analyzing the ROC curves of these indexes. We have found that resistin, RBP4 and MCP-1 levels appeared to be potential indicators for T2DM (AUC = 0.75, 0.78 and 0.79; *P *< 0.0001, respectively) (Fig. 2). Interestingly, resistin/RBP4 relative ratio had a better diagnostic performance of T2DM compared to resistin and RBP4 levels alone (AUC = 0.81 vs. 0.75 and 0.78, respectively) (Fig. 2a). Similarly, the diagnostic performance of MCP-1/RBP4 relative ratio was better compared to that of MCP-1 and RBP4 levels alone (AUC = 0.83 vs. 0.78 and 0.79, respectively) (Fig. 2b). Although visfatin/MCP-1 and leptin/MCP-1 indexes were correlated with several clinical parameters of T2DM, the diagnostic performance of these indexes were not better when compared with MCP-1 levels (AUC = 0.71 and 0.64 vs. 0.79, respectively) (Fig. 2c, d). In addition, we also examined the diagnostic potential of resistin/MCP-1, resistin/RBP4, visfatin/MCP-1, visfatin/SFRP5 and MCP-1/RBP4 indexes in insulin resistance/sensitivity and β-cell function. We have found that resistin/RBP4 and MCP-1/RBP4 indexes appeared to be potential indicators for β-cell function when compared to HOMA-β (AUC = 0.76 and 0.74, respectively) (Fig. 3a, b). Similarly, leptin/MCP-1 index could be an additional indicator for insulin resistance/sensitivity when compared to HOMA-IR (AUC = 0.72) and QUICK (AUC = 0.73) (Fig. 3c, d). These results indicate that the relative ratios of adipokines, especially resistin/RBP4 and MCP-1/RBP4 indexes, may also be considered as potential indicators for T2DM, insulin resistance/sensitivity and β-cell function.

## Discussion

Adipose tissue-derived cytokines play a significant role in the pathogenesis of inflammation and metabolic disorders such as obesity and T2DM [21, 30, 31]. Our previous study has shown that the levels of adiponectin and several pro-inflammatory cytokines were modulated in patients with T2DM compared to individuals without T2DM [24]. Similarly, we show in the present study that the levels of RBP4 and visfatin are increased while the MCP-1 and SFRP5 levels are decreased in patients with T2DM compared to control group. Although not all the comparisons reached the statistical significance, the levels of studied adipokines are modulated in individuals with overweight compared to those without. Our results revealed that the levels of studied adipokines including leptin, resistin, visfatin, SFRP5, MCP-1, and RBP4 are correlated with clinical parameters of overweight and T2DM. In addition, the relative ratios of studied adipokines are correlated with fasting glucose and HbA1c levels as well as with insulin resistance/sensitivity indexes such as HOMA-IR, QUICKI and β-cell function index (HOMA-β). These results indicate that adipose-derived cytokines are involved in the pathogenesis of obesity and T2DM and are associated with clinical outcomes of overweight and T2DM, and thus may be used as indicators of insulin resistance and β-cell function.

Leptin has been shown to play an important role in the pathogenesis of atherosclerosis, cardiovascular disease, inflammation, obesity and T2DM [32]. The biological role of leptin is to regulate appetite, hunger, body temperature and expenditure of energy through the hypothalamus [30, 31]. A recent study showed that lower leptin is associated with T2DM in obese adolescents [33]. However, leptin levels are not significantly different between elderly patients with T2DM and age-matched individuals without T2DM as shown in this study. This contradictory result might be due to the higher age of study subjects in our study. In addition, although leptin levels are not significantly correlated with metabolic parameters of obesity and T2DM, strong correlations of leptin levels with insulin levels and with indexes of insulin resistance/sensitivity and β cell function. These results suggest that leptin can be a promising indicator of insulin production and its resistance.

In line with a previous finding that resistin serum levels were significantly correlated with the inflammatory chemokines such as MCP-1 and RBP4, which are major players in the pathogenesis of metabolic syndromes [34]. A decreased resistin concentration by knocking out the resistin encoding gene or blocking resistin by antibody could protect against obesity-associated hyperglycemia, which is mainly due to recovering the response of the liver to insulin [35]. However, visfatin levels are not affected by the treatment of T2DM with basal insulin [36]. Although the difference is not significant between patients with and without T2DM in our study, resistin levels are increased in patients with overweight T2DM compared to those with non-overweight. These results are similar to other studies indicating that resistin levels are higher in patients with overweight T2DM compared to controls [37, 38]. Similarly, our results of visfatin levels in individuals with and without T2DM are consistent with several previous studies showing that visfatin levels are increased in individuals with overweight and T2DM compared to controls [39, 40]. Visfatin serum levels are significantly correlated with the accumulation of white adipose tissue (WAT), and visfatin expression was increased during the differentiation of adipocytes and according to the destruction of β cells [41, 42]. The negative correlation between the levels of visfatin and glucose indicates that visfatin is an important indicator for the development of obesity and related T2DM.

Similar to leptin, MCP-1 is significantly involved in the pathogenesis of several metabolic disorders including inflammation, obesity and diabetes [43]. MCP-1 also has chemotaxis activity that can promote leukocytes to leave the circulation and form the foam cells that subsequently lead to arterial plaque. Therefore, elevated level of MCP-1 production is a risk factor for the development of cardiovascular disease in patients with obesity-related T2DM [44]. Our study showed that the MCP-1 levels are decreased in patients with T2DM compared to healthy individuals, but increased in overweight healthy individuals compared to those with non-overweight. The strong correlations of MCP-1 levels with the levels of fasting glucose and HbA1c support the fact that MCP-1 contributes to increasing obesity-induced insulin resistance, the effect of macrophage infiltration into adipose tissue and elevated hepatic triacylglycerol [44]. These results suggested that increased MCP-1 production in adipose tissue caused by macrophage infiltration into adipose tissue leads to an increase of insulin resistance and consequently leads to T2DM [43]. Additionally, MCP-1 levels may be considered as a promising indicator for β-cell function and insulin sensitivity.

SFRP5 serum levels are correlated with markers of obesity (e.g. BMI, waist-hip ratio, percentage of body fat), and T2DM (e.g. insulin resistance and disorders of lipid metabolism) [45]. Our study showed that the SFRP5 levels are significantly increased in control group with overweight compared to those without overweight and are correlated with levels of fasting glucose, LDL-C and HOMA-β. This observation supports a recent study showing the association between *Sfrp5* gene expression and fat deposition in healthy adipose tissue [18]. Lower SFRP5 levels observed in patients with T2DM compared to controls in the current study is in accordance with a previous study showing the SFRP5 levels in Chinese patients [46]. However, these results are contradictory to a previous study showing that SFRP5 levels are elevated in Spanish patients with T2DM compared to prediabetic subjects and controls [19]. The association between SFRP5 levels and HOMA-β index clearly indicates the association of SFRP5 with β-cell function [17]. In addition, SFRP5 is sensitive to nutritional therapy suggesting the use of bioactive molecule as a biomarker for anti-inflammatory effects of diet [47].

Corroborating with previous studies [48, 49], our results also indicate that the RBP4 levels are significantly increased in patients with T2DM compared to controls. RBP4 has been shown to be associated with insulin resistance, visceral fat distribution, dyslipidemia and diabetes [22, 50]. The strong correlations of RBP4 levels with the levels of fasting glucose and HbA1c and HOMA-β index as well as the correlation of relative ratios based on RBP4 levels with QUICKI and HOMA-β indexes suggest that RBP4 plays an important role in β-cell dysfunction and has a potential diagnostic value for T2DM. RBP4 serum levels have a potential to be an indicator of insulin resistance and decreasing the RBP4 serum levels may be considered as one of the strategies for anti-diabetic therapies of overweight-related T2DM.

In addition, a previous study has proposed a novel index based on the relative proportion of adiponectin-to-resistin to predict insulin resistance [29]. The relative ratios of adiponectin/TNF-α, adiponectin/IL-1β, adiponectin/IL-10, TNF-α/IL-10 and IL-1β/IL-10 have been shown to be strongly correlated with the insulin resistance/sensitivity indexes (HOMA-IR and QUICKI) [24]. In this study, only the relative ratios of leptin/MCP-1 and MCP-1/RBP4 are correlated with resistance/sensitivity indexes. Nevertheless, the relative ratios of resistin/RBP4, visfatin/MCP-1 and MCP-1/RBP4 could be used as indicators for T2DM as these relative ratios are strongly correlated with the levels of fasting glucose, HbA1c and HOMA-β index. Furthermore, ROC curve analyses demonstrate the diagnostic potential of resistin/RBP4 and MCP-1/RBP4 indexes for T2DM and β-cell function while leptin/MCP-1 index is an additional indicator for insulin resistance/sensitivity. However, a panel comprised of adipose tissue-derived cytokines should be systematically developed and optimized before using as a diagnostic indicator for screening and predicting the development of T2DM.

Although our data indicate a significant association of adipokine levels with overweight and T2DM, the study remains several limitations. It was designed as a cross-sectional study, which cannot determine causal relationships between adipokine levels and the development of overweight and T2DM. Another limitation could be the limited number of data points of visfatin, SFRP5 and RBP4 levels in overweight controls. This may lead to an unsuccessful detection of the modulation of these adipokines in individuals with overweight and may weaken the findings that adipose tissue-derived cytokines play a vital role during the development of obesity and T2DM as well as reduce their diagnostic value for insulin resistance/sensitivity and β-cell function.

## Conclusions

Our study showed that the levels of leptin, resistin, visfatin, SFRP5, MCP-1, and RBP4 are significantly modulated during the development of overweight and T2DM and are correlated with clinical parameters of overweight and T2DM. The relative ratios of studied adipokines (e.g. resistin/RBP4, leptin/MCP-1 and MCP-1/RBP4) are correlated with fasting glucose and HbA1c levels as well as with insulin resistance/sensitivity and β-cell function indexes. Adipose-derived cytokines play important roles in the pathogenesis of obesity and T2DM and may serve as a prognostic marker to predict overweight-related T2DM.

## Abbreviations

T2DM: type 2 diabetes mellitus
BMI: body mass index
WHR: waist-to-hip ratio
GOT: glutamic-oxaloacetic transaminase (AST)
GPT: glutamic-pyruvic transaminase (ALT)
HDL-C: high-density lipoprotein—cholesterol
LDL-C: low-density lipoprotein—cholesterol
HOMA-IR: Homeostasis Model Assessment-Insulin Resistance
QUICKI: Quantitative Insulin Sensitivity Check Index
HOMA-β: homeostatic model assessment-β-cell function
